# The Velvet Underground Emerges

**DOI:** 10.1371/journal.pbio.1001751

**Published:** 2013-12-31

**Authors:** Roland G. Roberts

**Affiliations:** Public Library of Science, Cambridge, United Kingdom

Like all life on Earth, animals and fungi share a common ancestor, but it's been a good 1,500,000,000 years since we parted company, and that's been plenty of time for us to figure out different ways of going about things. Whether we're a mildew or a marmoset, a chanterelle or a chanticleer, a toadstool or a toad, we all need to respond to the outside world by using regulatory systems that turn environmental inputs into intracellular action. Typically, this chain of command might involve receptors at the cell membrane, followed by a cascade of cytoplasmic components that ultimately modulate a transcription factor in the nucleus, turning on or turning off genes.

The inevitable anthropocentrism of scientific research means that while we've thoroughly researched the pathways by which this is achieved in the animal kingdom, other parts of the tree of life have been less lucky. The fungal kingdom, which is thought to comprise well over a million species, hasn't fared too badly, so it's surprising to find that a key family of fungal regulator proteins—the “velvet” proteins—has had to wait until the publication of two recent *PLOS Biology* articles to reveal their basic function. The first one was published in July by Sinem Beyhan, Anita Sil, and colleagues, and the second one in the current issue by Yasar Luqman Ahmed, Ralf Ficner, and co-workers.

The first “velvet” protein was discovered by researchers who were investigating how light affects the decision of the filamentous mould *Aspergillus nidulans* to form sexual spores (conidia). The curious name comes from the role played by the VeA “*velvet*” gene in determining the developmental state of *Aspergillus*. Common lab strains had a point mutation in the veA gene, allowing these strains to produce more conidia than wild-type, thereby giving Petri plates inoculated with the fungus a characteristic velvety appearance.

The “velvet” proteins occur across the major fungal phyla (Ascomycota, Basidomycota), with many species having several different ones (*A. nidulans*, for example, has four—VeA, VelB, VelC, and VosA). They each share a protein domain of about 150 amino acids (the so-called “velvet domain”) with founding member VeA, and have been shown to be crucial for regulating reproduction and defensive mechanisms in response to stress. Some of these velvet-regulated responses involve the production of antibiotics or toxins, or enhancing a fungus' prowess as a pathogen of humans or our food crops, so the velvet proteins are of direct interest to us.

In the first article, Beyhan et al. [1] studied a soil-dwelling fungus, *Histoplasma capsulatum*. Like *Aspergillus*, this takes a filamentous form in the soil, but if it gets into a human body it turns nasty, assuming a pathogenic yeast-like form and causing a lung disease called histoplasmosis—common in AIDS patients because of their compromised immune system. The filament/yeast transition, which occurs in response to the raised temperature experienced in the lung, was known to be governed by three proteins, two of which are from the velvet family—Ryp2 and Ryp3 (the *Histoplasma* counterparts to *Aspergillus* VosA and VelB, respectively).

These authors showed that Ryp2 and Ryp3 physically interact with each other (and with a third protein, Ryp1—a transcription factor of the WOPR family) and specifically bind to the regulatory regions (promoters) of hundreds of genes across the *Histoplasma* genome. As a result, many genes are upregulated in response to the higher temperature of 37°C endured by the fungus within the human body. One of these genes encodes a fourth regulatory protein, Ryp4—a binuclear zinc cluster transcription factor—that is also required for this transcriptional circuit. The authors went on to identify a DNA sequence that is enriched in the genomic regions that associate with Ryp2 and Ryp3, and showed that these two velvet proteins can bind DNA directly when both proteins are incubated together with this target sequence in vitro. Taken together, these results strongly suggest that velvet proteins are themselves transcription factors that lack a previously recognised DNA-binding domain.

In the second paper, Ahmed et al. [2] turn back to *Aspergillus* to study the structure and function of its four velvet proteins. These not only regulate sexual and asexual reproduction, as mentioned above, but also the synthesis of aflatoxin (a powerful toxin and carcinogen) and trehalose (a sugar that protects the fungus against a variety of environmental stresses). The authors confirm that the *Aspergillus* velvet proteins bind specifically to a genomic DNA sequence that shows some similarity to that bound by their *Histoplasma* equivalents in Beyhan's paper. They then crystallise the velvet domain of VosA and determine its atomic structure.

**Figure 1:**
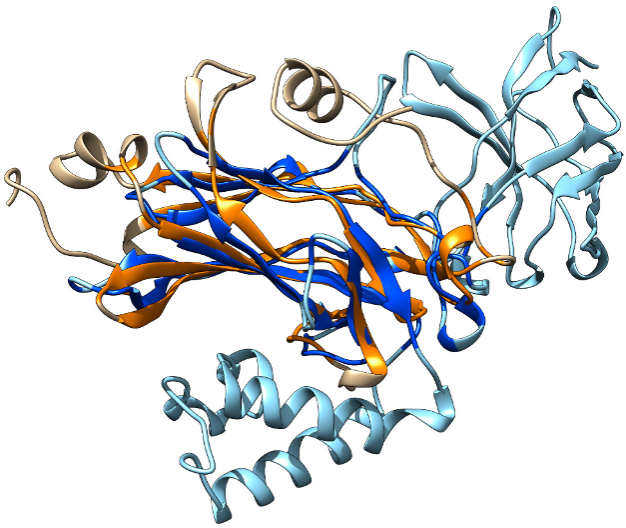
The *Aspergillus* VosA velvet domain (beige and orange) partially superimposed on the RHD from human NF-κB (pale and dark blue) to show the structural similarity between these transcription factors from different kingdoms. *Image Credit:* Fig. S8B from Ahmed et al.: PLoS Biol 11(12): e.1001750. doi:10.1371/journal.pbio.1001750.

This reveals two molecules of VosA bound together intimately in a homodimer, and although the velvet domain as a whole has a novel architecture, its detailed structure shows an unanticipated similarity to a very familiar protein family indeed. A twisted seven-stranded “β-sandwich” comprising two-thirds of the amino acids in the velvet domain can be neatly superimposed on part of the structure of the Rel homology domain (RHD; see Figure 1). The RHD has only been found in transcription factors from the animal kingdom, including a lynchpin of the mammalian immune system, NF-κB. This structural similarity between fungal velvet domains and animal RHDs hadn't been spotted previously because the similarity between their raw amino acid sequences is barely discernible.

Guessing that VosA might bind DNA in a similar way to NF-κB, Ahmed *et al.* used the known structure of NF-κB's complex with DNA to predict which amino acids of VosA might be involved in recognising DNA sequences. They mutated these and found that the mutant VosA no longer bound to its specific recognition site and worked poorly in *Aspergillus* cells, confirming that velvet proteins may well bind DNA in a similar way to RHD proteins like NF-κB. Because velvet proteins often collaborate with each other to regulate responses, the authors also determined the structure of the VosA-VelB heterodimer.

What are we to make of the surprising resemblance between these two protein domains—RHD and velvet—from two kingdoms separated by a billion and a half years of evolution? Is it an example of independent solutions to a common problem—are there only so many ways for a protein to bind to DNA, and these two protein families have stumbled upon the same answer? Or has deep time disguised a common origin—did our common Opisthokont ancestor in some dismal ancient pond carry a crucial regulatory protein that retained a common structural core but later embellished it in different ways? Ahmed et al. lean towards the latter, drawing together a set of structural, functional and phylogenetic arguments for a common ancestral velvet/RHD protein whose descendants have gone on to greater things in two kingdoms of life.

Between them, these two papers make a clear structural and functional case that the velvet proteins are *bona fide* transcription factors, and that the trademark velvet domain mediates specific interactions with DNA and with other velvet proteins—a solid basis for future work on this crucial family of regulators.

**Ahmed YL, Gerke J, Park H-S, Bayram Ö, Neumann P, et al. (2013) The Velvet Family of Fungal Regulators Contains a DNA-Binding Domain Structurally Similar to NF-κB.** doi:10.1371/journal.pbio.1001750.
